# Development of two shortened systematic review formats for clinicians

**DOI:** 10.1186/1748-5908-8-68

**Published:** 2013-06-14

**Authors:** Laure Perrier, Nav Persaud, Anita Ko, Monika Kastner, Jeremy Grimshaw, K Ann McKibbon, Sharon E Straus

**Affiliations:** 1 Li Ka Shing Knowledge Institute, St Michael’s Hospital, 30 Bond Street, Toronto, ON M5B 1W8, Canada; 2 Continuing Education & Professional Development, Faculty of Medicine, University of Toronto, 500 University Avenue, Toronto, ON M5G 1V7, Canada; 3 Department of Family and Community Medicine, University of Toronto, 500 University Avenue, Toronto, ON M5G 1V7, Canada; 4 Mechanical and Industrial Engineering, University of Toronto, 5 King’s College Road, Toronto, ON M5S 3G8, Canada; 5 Department of Medicine, Clinical Epidemiology Program, Ottawa Hospital Research Institute, University of Ottawa, 501 Smyth Road, Box 711, Ottawa, ON K1H 8L6, Canada; 6 Health Information Research Unit, Dept of Clinical Epidemiology and Biostatistics, McMaster University, CRL Building, 1280 Main Street West, Hamilton, ON L8S 4K1, Canada

**Keywords:** Review literature as topic, Evidence-based medicine

## Abstract

**Background:**

Systematic reviews provide evidence for clinical questions, however the literature suggests they are not used regularly by physicians for decision-making. A shortened systematic review format is proposed as one possible solution to address barriers, such as lack of time, experienced by busy clinicians. The purpose of this paper is to describe the development process of two shortened formats for a systematic review intended for use by primary care physicians as an information tool for clinical decision-making.

**Methods:**

We developed prototypes for two formats (case-based and evidence-expertise) that represent a summary of a full-length systematic review before seeking input from end-users. The process was composed of the following four phases: 1) selection of a systematic review and creation of initial prototypes that represent a shortened version of the systematic review; 2) a mapping exercise to identify obstacles described by clinicians in using clinical evidence in decision-making; 3) a heuristic evaluation (a usability inspection method); and 4) a review of the clinical content in the prototypes.

**Results:**

After the initial prototypes were created (Phase 1), the mapping exercise (Phase 2) identified components that prompted modifications. Similarly, the heuristic evaluation and the clinical content review (Phase 3 and Phase 4) uncovered necessary changes. Revisions were made to the prototypes based on the results.

**Conclusions:**

Documentation of the processes for developing products or tools provides essential information about how they are tailored for the intended user. One step has been described that we hope will increase usability and uptake of these documents to end-users.

## Background and significance

Systematic reviews are one tool available to clinicians that provide the current best evidence. Ideally, authors of systematic reviews employ rigorous methods to select credible and relevant information to generate summative reports [1-3]. Although systematic reviews are identified as providing the best evidence for a clinical question [2,3], the literature indicates that they are not being used regularly for healthcare decision making [4,5]. One proposed solution is to create filtered resources, where the included original studies and reviews have been subject to explicitly formulated methodological criteria [6]. An example of this is ACP Journal Club (acpjc.acponline.org). This allows information to be validated and refined to facilitate rapid reading [7] by clinicians, whose time constraints are a significant challenge in keeping up to date with current research [8].

Several clinical information tools currently exist that present information from systematic reviews in a shortened or summarized manner (such as the BMJ PICO abridged research articles). We completed two comprehensive reviews of the literature that examined the impact of interventions for seeking, appraising, and applying evidence from systematic reviews in decision-making by clinicians or policymakers [9,10] and specifically screened for studies that evaluated different strategies for presenting a systematic review. We located two trials using GRADE (Grading of Recommendations Assessment, Development and Evaluation) by Rosenbaum and colleagues [11,12] who examined a ‘summary of findings’ table added to Cochrane systematic reviews. They reported that participants found it easier to locate results for important outcomes, were more likely to correctly answer questions regarding results, and spent less time finding key information. However, it is necessary to be thoughtful in interpreting these results, as small samples were used and participants were drawn from a convenience sample, including those who had an affiliation with the Cochrane Collaboration. Aside from these two trials that demonstrate considerable limitations related to study quality, the studies screened and reviewed revealed no literature in either guiding the creation of different formats or rigorously evaluating the impact on end-users.

**Figure 1:**
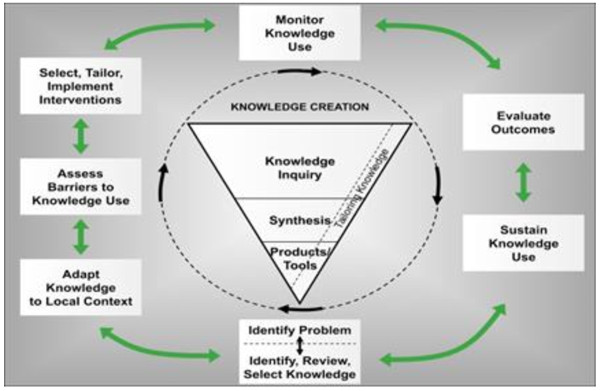
Knowledge to action (KTA) framework.

The development of the shortened systematic review formats is informed by the Knowledge-to-Action Cycle (Figure 1) proposed by Graham and colleagues [13]. At the centre of the Knowledge-to-Action cycle is the ‘knowledge funnel,’ which focuses on the process through which knowledge is refined, distilled and tailored to the needs of end-users such as healthcare professionals. Knowledge tools and products are identified as ‘third-generation knowledge’ and consist of knowledge synopses that present knowledge in a clear, concise and user-friendly format. An over-arching component to the knowledge funnel is tailoring knowledge, and this process begins well before seeking the input from end-users. Of key importance is the rigor and methodical process taken beforehand that uses evidence and conventional standards to create knowledge tools, rather than relying exclusively on methods such as consulting with colleagues and experts to gain opinions for the inclusion of content. Documenting the development of products or tools with this evidence-based approach gives critical information about the process of tailoring tools for the intended user. Providing details lends support to the development of interventions in a rigorous, thoughtful manner before implementation, and allows for the concise capturing and sharing of key concepts, plans and processes.

### Objective

To describe the development process of two shortened formats for a systematic review intended for use by primary care physicians as an information tool for clinical decision-making.

## Methods

We employed a series of strategies to create two shortened formats, case-based and evidence-expertise, to aid in decision-making for clinicians before seeking input from users on their preferences. The components of the process included:

1. selection of a systematic review and creation of initial prototypes that represent a shortened version of the systematic review;

2. a mapping exercise to identify obstacles described by clinicians in using clinical evidence in decision making;

3. a heuristic evaluation (a usability inspection method); and

4. a review of the clinical content in the prototypes.

### Phase 1: selecting a systematic review and creation of initial prototypes

We chose a full-length systematic review to be used for developing prototypes by having four generalist clinicians select from a list of systematic reviews that were drawn from 120 medical journals published in the last five years on topics relevant to primary care [14]. These physicians are chosen from a pool of more than 4,000 physicians who have received formal training in rating articles to identify those that are pertinent to practice as part of a larger program in evidence-based health informatics at McMaster University, Canada [14]. The clinicians were asked to rate the articles that they believed would be important to practicing primary care clinicians using the McMaster PLUS (Premium Literature Service) 7-point Likert scale, where 1 indicates that the article is definitely not relevant and 7 indicates that it is directly and highly relevant. The PLUS scale is used by the Health Information Unit at McMaster University to identify articles for inclusion in a secondary journal (ACP Journal Club) and BMJ Updates [14]. The Health Information Unit supplied a list of 927 systematic reviews that scored 6 or better (out of 7) on the McMaster PLUS scale. Initially, two physicians (one internal medicine physician and one family physician) reviewed all the systematic reviews supplied and independently voted on the three most relevant to generalist physicians. The final review was selected by a third family physician independently. The systematic review that was selected for this study was: ‘Systematic review of rosacea treatments.’ van Zuuren EJ, Gupta AK, Gover MD, Graber M, Hollis S. Journal of the American Academy of Dermatology. 2007 Jan;56(1):107–15.

Two shortened formats were developed in collaboration with a human factors engineer using the selected systematic review. Human factors is the application of what is known about human capabilities and limitations to the design of tools in order to enable more effective use [15]. Guiding principles for user-centered design were employed, which focuses on making tools that are usable, useful and accessible for the development of the prototypes [16]. The initial prototypes were designed to be one page in length (front and back), giving them the flexibility to be viewed online (as a PDF document) or be printable. Once this is finalized, our future plans include optimizing them for handheld environments. The decision for this sequencing was based on the lack of evidence that increased availability and advances in electronic health technology affect the use of evidence in practice [17]. The first format used a case study to present contextualized information (case-based format), and the second format integrated evidence and clinical expertise (evidence-expertise format). The case-based format was designed to provide evidence within the context of a specific situation, presenting a real-world example of how the evidence could be used in decision-making. This format was chosen since text is easier to understand when it has personalized elements including examples, such as case studies [18-22]. Personalized texts prompt readers to recall more information [21,22], as well as allow instructions and information to be embedded more succinctly [23]. The evidence-expertise format was guided by David Sackett’s definition of evidence-based medicine, highlighting the integration of clinical expertise and the best external evidence [24]. El Dib and colleagues [25] analyzed 1,016 randomly selected systematic reviews covering a wide variety of clinical topics and found that approximately half reported results that neither supported nor refuted the intervention tested. Similarly, less than 5% of 2,535 Cochrane systematic reviews explicitly state that no more research is needed or feasible [26]. Primary care physicians expressed the need to have an explicit statement about where the evidence was absent and how clinical expertise could bridge this gap when describing their preferences for the presentation of evidence [27]. These findings indicate that supplementing the review with clinical expertise may be useful, since finding a systematic review relating to a clinical question does not assure guidance for a clinical decision. Content was developed for the case study in the case-based format, and information was obtained specifically to present an expert interpretation for the evidence-expertise format. All other information presented in the shortened formats was drawn directly from the original full-text systematic review.

### Phase 2: mapping exercise

The aim of the mapping exercise was: to identify the intrinsic obstacles (*i.e*., specific to the information tool or document) to answering doctors’ questions about patient care with evidence; and to identify at least one attribute within each shortened systematic review format (case-based and evidence-expertise) that addresses these obstacles. The mapping exercise was not intended to provide a guarantee that each obstacle had been eliminated from the prototypes, but served as a methodical inspection of the documents, with the intrinsic obstacles as guidance for identifying at least one instance where they had been addressed.

#### Identifying intrinsic obstacles

Ely and colleagues extensively studied the information needs of family physicians [8,28-34]. They used this work to develop a taxonomy of 59 obstacles encountered while searching for evidence-based answers to doctors’ questions (Additional file 1) [8]. With regards to information tools or documents, the 59 obstacles cover both extrinsic factors (*e.g*., a physician does not have a computer in his or her office to search for information), and intrinsic factors (*e.g.*, the wording of a clinical practice guideline is too vague). In our study, two people (LP, NP), an information specialist and a family physician, independently reviewed each obstacle and identified if it was an intrinsic factor of an information tool or document, or an extrinsic factor. The intrinsic obstacles are the elements that have the potential to be addressed in the development of an information tool or document. Discrepancies were resolved by discussion until consensus was reached.

#### Linking items in shortened reviews that address intrinsic obstacles

We reviewed both formats (case-based and evidence-expertise) to determine if they addressed obstacles identified as intrinsic factors. If intrinsic obstacles were not addressed, we changed the documents. For example, if the obstacle ‘resource not authoritative or trusted’ was not addressed, the citation (including authors and journal name) for the systematic review would be added.

### Phase 3: completing a heuristic evaluation

Heuristic evaluation is a usability inspection method that involves the examination of the prototypes by comparing them to recognized usability principles (the ‘heuristics’) [16]. It is used to identify major usability problems of a product or tool in a timely manner with reasonable cost [35-38]. Having a number of heuristic evaluators will identify more usability problems; however, it is recommended that a cost-benefit consideration be employed to determine the number of evaluators appropriate for an individual project [39]. Since the prototypes for this study were undergoing a multi-step development and evaluation process, we decided to use one heuristic evaluator. The consultant who carried out the evaluation had no involvement in the study. She was selected to conduct this phase as she has a PhD in mechanical and industrial engineering, and conducts research related to the science and technologies of human factors [40]. A modified set of heuristics applicable to the analysis of printed materials (based on the tool provided by Nielsen [41]) was used for the heuristic evaluation (Additional file 2). As per the heuristic evaluation methodology by Nielsen [42], the errors are first identified, then classified by severity, *i.e*., cosmetic, minor, moderate, major, or catastrophic. The severity estimates are based on frequency, impact, and persistence of errors.

### Phase 4: reviewing the clinical content

Clinical content was reviewed by a family physician (NP). The role of the clinical content reviewer was to ensure that the information was transferred from the original document to the shortened versions accurately (and not to evaluate the accuracy or quality of the information) [43]. The clinical content reviewer is an independently licensed and active family physician with three years of clinical experience. He was selected based on clinical knowledge and willingness to volunteer time to this study. One reviewer was sufficient, as the function of the exercise was to identify obvious errors, and this was done with the knowledge that the next step in development would be to assess the prototypes using iterative focus groups with end-users (not described in this paper).

## Results

### Phase 1: selecting a systematic review and creation of initial prototypes

We developed and refined a summary of a systematic review on rosacea in two formats, case-based and evidence-expertise, which addressed many obstacles clinicians encounter while searching for evidence-based answers to questions. As reported in the Methods section, we selected a systematic review of rosacea treatments and developed summaries in case-based and evidence-expertise formats.

### Phase 2: mapping exercise

#### Identifying intrinsic obstacles

Thirty-two of 59 factors from Ely’s framework were indicated as intrinsic to an information tool. The strength of agreement between the two reviewers (LP, NP) was very good (kappa statistic of 0.82; CI: 0.687 to 0.972) [44]. Ely and colleagues organized the obstacles into five categories [8]. The majority of the intrinsic obstacles (26 of 32; 81%) in our study fell under the third category, ‘searching for relevant information to answer a question about patient care.’ Four out of the 32 obstacles were categorized as being relevant to ‘formulating an answer,’ and the final two were relevant to ‘using the answer to direct patient care.’

#### Linking items in shortened reviews that address intrinsic obstacles

Eight items from Ely’s framework could not be addressed, as they were not applicable to the mapping. For the 24 items that were applicable, five were identified as being absent from one of the formats. For instance, both formats did not address the obstacle ‘failure to define important terms*.*’ This prompted the addition of a definition of odds ratio to the case-based format, given the supporting evidence that statistics commonly found in medical journals are not readily understood by clinicians [45-48]. The decision was made to add this to only one shortened format, since the next tool development step will be to run focus groups to gain input from end-users. The focus groups will provide the opportunity to determine if users perceived ‘odds ratio’ as an important term or as an unnecessary feature. All other intrinsic obstacles centered around the information being up-to-date, relevant, and authoritative or trustworthy. These issues were resolved by adding the full citation, along with the objectives of the study to the evidence-expertise format. For some of the intrinsic obstacles identified, it was not possible to find evidence to support how the prototype could be changed to address the obstacle. As an example, for the obstacle ‘resource not authoritative or trusted,’ this can be addressed explicitly in the review by including the citation for the original publication. In contrast, for the obstacle ‘resource is poorly organized,’ we searched the literature for a systematic review that offered evidence of designing informational text to make linkages with the best evidence available; however, none was found. For these obstacles, it was only possible to identify single studies and present this as supportive evidence from the literature. For example, the obstacle ‘resource is poorly organized’ was addressed by using titles and headings, and identifying literature that links this to better recall and comprehension for users [49-52]. Table 1 indicates if the intrinsic obstacle was addressed or not, as well as identifying items that were not applicable. An Additional file 3 lists comprehensive descriptions of how they were addressed if relevant, and actions taken if obstacles were not initially available in the prototypes.

**Table 1:** Mapping of intrinsic obstacles to items on prototypes

**Item**		**Format**	**Yes**	**No**	**Not applicable**
1	Topic or relevant aspect of topic not included in a resource that should logically include it	Evidence-Expertise		**X**	
		Case-Based	**X**		
2	Inadequacy of the resource’s index	Evidence-Expertise			**X**
		Case-Based			**X**
3	Resource poorly organized	Evidence-Expertise	**X**		
		Case-Based	**X**		
4	Resource not clinically oriented	Evidence-Expertise	**X**		
		Case-Based	**X**		
5	Resource not authoritative or not trusted	Evidence-Expertise		**X**	
		Case-Based	**X**		
6	Resource not current	Evidence-Expertise	**X**		
		Case-Based	**X**		
7	Inability to interact with a general resource as one could with a human resource	Evidence-Expertise			**X**
		Case-Based			**X**
8	Incorrect information	Evidence-Expertise	**X**		
		Case-Based	**X**		
9	Information not current	Evidence-Expertise		**X**	
		Case-Based	**X**		
10	Failure to anticipate ancillary information needs	Evidence-Expertise			**X**
		Case-Based			**X**
11	Failure to address common comorbid conditions	Evidence-Expertise			**X**
		Case-Based			**x**
12	Inadequate differential diagnosis	Evidence-Expertise			**X**
		Case-Based			**X**
13	Failure to define important terms	Evidence-Expertise		**X**	
		Case-Based		**X**	
14	Inadequate description of clinical procedures	Evidence-Expertise			**X**
		Case-Based			**X**
15	Vague or tangential information	Evidence-Expertise	**X**		
		Case-Based	**X**		
16	Unnecessarily cautious writing style	Evidence-Expertise	**X**		
		Case-Based	**X**		
17	Tertiary care approach to primary care problem	Evidence-Expertise	**X**		
		Case-Based	**X**		
18	Biased information due to conflicts of interest	Evidence-Expertise	**X**		
		Case-Based	**X**		
19	Failure to address the clinical question	Evidence-Expertise	**X**		
		Case-Based	**X**		
20	Failure to study the comparison of interest	Evidence-Expertise	**X**		
		Case-Based	**X**		
21	Failure to study the outcome of interest	Evidence-Expertise	**X**		
		Case-Based	**X**		
22	Failure to study the population of interest	Evidence-Expertise	**X**		
		Case-Based	**X**		
23	Evidence based on flawed methods	Evidence-Expertise	**X**		
		Case-Based	**X**		
24	Failure to cite or include relevant evidence	Evidence-Expertise	**X**		
		Case-Based	**X**		
25	Inadequate synthesis of multiple bits of evidence	Evidence-Expertise			**X**
		Case-Based			**X**
26	Difficulty applying results of randomized clinical trials to individual patients	Evidence-Expertise			**X**
		Case-Based			**X**
27	Failure to directly or completely answer the question	Evidence-Expertise	**X**		
		Case-Based	**X**		
28	Answer too long or too short	Evidence-Expertise			**X**
		Case-Based			**X**
29	Answer directed at the wrong audience	Evidence-Expertise	**X**		
		Case-Based	**X**		
30	Difficulty addressing unrecognized information needs apparent in the question	Evidence-Expertise	**X**		
		Case-Based	**X**		
31	Answer not trusted	Evidence-Expertise		**X**	
		Case-Based	**X**		
32	Answer inadequate	Evidence-Expertise	**X**		
		Case-Based	**X**		

### Phase 3: heuristic evaluation

The heuristic evaluation indicated that there were no major usability problems. Several moderate usability issues were identified, including wording that could potentially confuse readers, the placement of information (*e.g.*, an evidence rating appearing in different columns of tables), or omissions that could potentially confuse readers (*e.g.*, no evidence ratings for some treatments). Minor issues concerned the small size of text and layout for the case-based format. We used all feedback to modify the prototype formats.

### Phase 4: clinical content review

The clinical content review revealed that the evidence-expertise format accurately reflected the information in the full-length review. One issue was detected in the case-based prototype, and the reviewer recommended modifications to the case that included not focusing on iatrogenic rosacea, removing references to prednicarbate, and using the term ‘family physician’ instead of ‘general practitioner.’ All of these changes were made to the case-based prototype. Additional file 4 and Additional file 5 provide the prototypes before and after the mapping exercise, heuristic evaluation, and clinical content review.

## Discussion

We have described the components of the development process for two shortened formats of systematic reviews. Aside from the first phase of selecting and creating the initial prototype, each component of the development process stimulated alterations within the two formats. The second phase was mapping items within the prototypes to obstacles identified by doctors they encountered while searching for evidence-based information, as described by Ely and colleagues [8]. Most obstacles were addressed within the prototypes, but some changes were prompted, such as adding the citation in order to address the obstacle ‘resource is not current.’ The heuristic evaluation and clinical content review stimulated additional modifications. None were significant, and the clinical content review prompted amending the content of the case study offered in one of the prototypes.

Although shortened formats may be familiar and currently available to clinicians, no formal evaluations of these formats have been published. This was confirmed when the 8,104 relevant records of the published and gray literature from our systematic reviews were also examined for studies that described alternate formats [9,10]. No alternate formats concentrating on the presentation of systematic reviews that were developed, tested and evaluated in a rigorous manner for healthcare professionals were found in our literature review.

### Limitations

The development process for the prototypes described in this paper needs to be considered within the context of certain limitations. It may not be possible for all groups to collaborate directly with a human factors engineer when developing information tools. One consideration is to hire consultants for this expertise and include this cost into the budget of research grants. Alternatively, online resources can be used to provide guidance [16]. A single reviewer was used for both the heuristic evaluation and the clinical content review. Although using more than one reviewer has the potential to identify more problems, a pragmatic approach was taken, and cost-benefit considerations guided this decision. For the clinical content review, using one reviewer was also influenced by the fact that the full-length systematic review came from a peer-reviewed journal, which meant that the clinical content had already gone through peer review. We made these decisions with the knowledge that this process was the first step in a multi-step strategy in which the prototypes will be tested by end-users in a series of focus groups.

## Conclusions

Reporting these steps and the outcomes has made the process for the development of the two prototypes transparent for users and publishers. As well, it encompasses one step of developing a viable document, which we hope will increase its usability and uptake to end-users.

### Future development

In the next step in the development of these prototypes, we plan to conduct focus groups with primary care physicians to gain their input on the format, presentation and layout of the revised prototypes after all revisions had been made. The purpose of the focus groups is to generate essential components of the shortened systematic reviews, and to seek reactions to these prototypes and their potential for clinical decision making. This activity will provide the opportunity to hear from users about their requirements when using such tools, as well as to make changes and correct problems as they emerge. Iterative focus groups allow the chance to take results and quickly incorporate them into the new design. This is an important step of the Knowledge-to-Action cycle that facilitates the tailoring of information tools to the needs of potential users [13]. Following this, we will complete usability testing. Finally, we will test the prototypes in a randomized trial to determine their impact on knowledge and ability to apply the evidence to clinical scenarios.

## Competing interests

Sharon E. Straus is an Associate Editor for Implementation Science. All editorial decisions regarding this manuscript were made independently by other editors. None disclosed for all other authors.

## Authors’ contributions

SES conceived of the idea. JG and KAM provided guidance and advice on the methods of the study. LP, MK and NP participated in the mapping exercise. NP conducted the clinical content review. LP and AK prepared the original prototypes. LP wrote the manuscript, and all authors provided editorial advice. All authors read and approved the final manuscript.

## Supplementary Material

Additional file 1 Obstacles to answering doctors’ questions about patient care with evidence. Click here for file

Additional file 2 Assessment criteria for heuristic evaluation: modified for analysis of print materials. Click here for file

Additional file 3 **Mapping exercise: Obstacles and descriptions of how they were addressed **[53,54]**.** Click here for file

Additional file 4 Shortened format: Initial versions of two prototypes. Click here for file

Additional file 5 Shortened format: Revised versions of two prototypes. Click here for file
