# Supplementary material for: Development of two shortened systematic review formats for clinicians
Source: Implement Sci. 2013 Jun 14;8:68. doi: 10.1186/1748-5908-8-68 (PMC3691647; doi:10.1186/1748-5908-8-68)
Supplement: Additional file 1 — Obstacles to answering doctors’ questions about patient care with evidence. [file 1748-5908-8-68-S1.doc]

**Additional File 1. Obstacles to answering doctors' questions about patient care with evidence**

Reference: Ely JW, Osheroff JA, Ebell MH, Chambliss ML, Vinson DC, Stevermer JJ, Pifer EA. BMJ. 2002;324(7339):710.

| Obstacles to answering clinical questions |
| --- |
| 1. Obstacles related to recognising an information need |
| 1.1 Doctor's lack of awareness of an information need.  The doctor makes decisions about patient care, completely unaware of a gap in knowledge. |
| 1.2 Doctor's suppression of a recognised information need.  On some level the doctor is aware of a gap in knowledge but suppresses it due to time pressures, embarrassment, personal characteristics, or characteristics of the clinical setting. |
| 2. Obstacles related to formulating the question |
| 2.1. Inability to answer patient specific questions with general resources.  Patient specific questions (“What is this rash?”) and vague cries for help (“I don't know what to do with this patient”) cannot be answered by a general resource. |
| 2.2. Missing patient data requiring unnecessarily broad search for information.  Questions that include demographic information, clinical information, and patient preferences may help focus the search and shorten the answer. The type of patient data that would help varies depending on the question and may not be apparent until the search for an answer is under way. |
| 2.3. Uncertainty about the scope of the question and unspoken ancillary questions.  It may not be clear whether the original question should be broadened to include potential ancillary questions. The answer to “What is the antibiotic of choice for pneumonia in a 5 year old?” could include ancillary prescribing information (for example, dose, adverse effects) thus avoiding the need to consult a second resource. An intermediary searcher may not anticipate such ancillary questions. |
| 2.4. Obstacles related to modifying the question |
| 2.4.1. Uncertainty about changing specific words in the question.  The doctor may ask a question using words that lead to difficulties in the search for information. For example, the word “sciatica” is less conducive to a literature search than “low back pain.” |
| 2.4.2. Unhelpful modifications resulting from flawed communication between the doctor and searcher.  The real information need may be lost as it is communicated from the doctor to an intermediary searcher, such as a librarian. |
| 2.4.3. Need for modifications apparent only after the search has begun.  Often a helpful modification to the original question becomes apparent only after the search for information is under way and the searcher learns more about the topic. |
| 2.4.4. Difficulty modifying questions to fit the PICO format (patient, intervention, comparison, outcome).  Sackett et. Al. suggest four elements for clinical questions: patient or problem, intervention, comparison, and outcome. However, many clinical questions do not involve interventions, comparisons, or outcomes. |

| 3. Obstacles related to seeking information |
| --- |
| 3.1. Failure to initiate the search |
| 3.1.1. Doubt about the existence of relevant information.  A search may not be initiated because the doctor doubts the existence of relevant information or doubts that any information found would change the plan of care. |
| 3.1.2. Question not important enough to justify a search.  For example, the work of searching may not be justified if the question was motivated more by curiosity than by patient care. |
| 3.1.3. Lack of time to initiate search.  Practising doctors have only a few minutes to answer their questions, but extensive time consuming searches are often required to adequately answer clinical questions. |
| 3.1.4. Ready availability of consultation which leads to a referral rather than a search. Practising doctors may refer patients to consultants if they believe excessive time and effort would be required to learn enough about the problem to feel comfortable managing it themselves. |
| 3.2. Obstacles related to the search for information |
| 3.2.1. Uncertainty about where to look for information.  It can be difficult to decide which resources will be most helpful and what should determine the selection of resources. Time available? Familiarity with resource? Type of question? |
| 3.2.2. Less than optimal strategy due to lack of searcher skill.  When the searcher lacks skills for searching the literature or lacks familiarity with the internet, relevant information may be missed. |
| 3.2.3. Uncertainty about which order to search resources.  After selecting potentially helpful resources it may not be obvious in which order to search these resources or what should guide the order (physical accessibility, resource quality, time available, etc). |
| 3.2.4. Uncertainty about narrowing the search without missing relevant information*.*  When faced with an overwhelming body of knowledge about a topic, it may not be clear how to narrow the search (for example, during a Medline search) without losing relevant information. |
| 3.2.5. Uncertainty about which articles to read thoroughly and how thoroughly to read them. It is often not clear how to select resources, such as journal articles, for thorough reading. Should the decision be based on the title, the abstract, the prestige of the journal, other factors? Once an article has been selected for more thorough reading, how thoroughly should it be read and how can the needed information be found without reading every word. |
| 3.2.6. Uncertainty about the adequacy of the search (when to stop searching).  Often it is not clear when to stop searching for information. When can the question be answered adequately? How can it be known that all the important evidence has been found? |
| 3.2.7. Uncertainty about the meaning of null search results.  If no interaction between two drugs is listed, does that mean no interaction exists? If a relevant article makes no mention of treatment for a disease, without explicitly stating that there is no treatment, does that mean there is no treatment? |

| 3.2.8. Inadequate indexing of databases used for computerised literature searching.  For example, a MeSH term for the topic of interest might not exist or a relevant article might not be indexed under the intuitive MeSH term. |
| --- |
| 3.2.9. Lack of time to search adequately.  Once initiated, the search for information may be suboptimal because pressures on time lead to a poor choice of resources. |
| 3.3. Obstacles related to knowledge resources (for example, books, journals, computers) |
| 3.3.1. Resource physically distant.  The resource may not be readily accessible to the doctor. |
| 3.3.2. Topic or relevant aspect of topic not included in a resource that should logically include it.  Based on the title of the article or book, coverage of the topic would have been expected. |
| 3.3.3. Inadequacy of the resource's index.  The topic may be covered in the text of a book or computer resource but not listed in the index. The topic may be listed in the index but not under an intuitive entry. The index may be inadequately cross referenced. |
| 3.3.4. Resource poorly organised*.*  Resources may be poorly organised within a personal library or reprint file. Information within a resource may be poorly organised or have inadequate titles or subtitles. |
| 3.3.5. Resource not clinically oriented.  For example, textbooks are often organised by disease rather than by clinical findings, which forces the doctor to “work backwards.” |
| 3.3.6. Obstacles related to computers (hardware and software problems).  Bugs, slowness, unintuitive software, Internet problems. |
| 3.3.7. Difficulty accessing resources in libraries.  The library may not hold the needed journal or book. The needed volume may not be on the shelf. Journal supplements are often difficult to find or missing. |
| 3.3.8. Resource not authoritative or not trusted.  The resource may not be authoritative or it may not be trusted by the searcher. |
| 3.3.9. Resource not current.  The resource may not be current or it may be difficult to know if it is current (for example, undated internet sites and printed material). |
| 3.3.10. Inability to interact with a general resource as one could with a human resource.  Most general resources do not allow real time interaction with the searcher as could happen with a human resource. There can be no follow up questions. |
| 3.4. Obstacles related to information within resources |
| 3.4.1. Incorrect information.  The information simply may be wrong. |
| 3.4.2. Information not current.  The resource containing the information may or may not be current, but the information itself is not current. |
| 3.4.3. Failure to anticipate ancillary information needs.  There is inadequate anticipation of likely ancillary or follow up questions (for example, the name of a recommended drug is provided but not the dose, forcing the searcher to consult another resource). |
| 3.4.4. Failure to address common comorbid conditions.  The information refers to patients in general but does not account for common comorbid conditions or the question is answered for patients with a comorbid condition but not for patients in general. |
| 3.4.5. Inadequate differential diagnosis.  A differential diagnosis consists of a list of diseases with little information about how to distinguish among the possibilities. |
| 3.4.6. Failure to define important terms. The information includes terms that are not defined. For example, treatment may vary depending on whether the disease is mild, moderate, or severe, but these terms are not defined. |
| 3.4.7. Inadequate description of clinical procedures*.*  A clinical procedure (for example, thoracentesis) is described but there is insufficient detail to allow the doctor to do it. |
| 3.4.8. Vague or tangential information.  The information does not allow the question to be answered directly because of a vague, tangential, or overly general format. |
| 3.4.9. Unnecessarily cautious writing style.  The information is overly cautious and may contain unnecessary hedge words (“can,” “may,” etc). The caution may be legitimate (inadequate evidence to support a definitive statement), but it may be unnecessary. |
| 3.4.10. Tertiary care approach to primary care problem.  Available information may take an urban interventionist tertiary care approach, which may not be useful to a rural primary care doctor with a non-interventionist philosophy. |
| 3.4.11. Biased information due to conflicts of interest.  The author or editor may have conflicts of interest. |
| 3.5. Inadequacy of available evidence |
| 3.5.1. Failure to address the clinical question.  Available studies have not adequately addressed the question (for example, “Is smoking a risk factor for sinusitis?”). |
| 3.5.2. Failure to study the comparison of interest.  Drug companies often sponsor clinical trials comparing drug A with placebo, but the question is whether drug A is better than drug B. |
| 3.5.3. Failure to study the outcome of interest.  An intermediate outcome, such as serum cholesterol level, may be studied rather than more clinically important outcomes, such as myocardial infarction or death. |
| 3.5.4. Failure to study the population of interest.  It may not be appropriate to apply results from a referral population to the primary care setting. |
| 3.5.5. Evidence based on flawed methods.  Multiple flaws (for example, selection bias, misclassification bias, confounding, etc) may invalidate the results. |
| 3.6. Obstacles related to the use of available evidence |
| 3.6.1. Failure to cite or include relevant evidence.  Evidence exists but is not cited. It may be difficult to know if evidence exists and, if it exists, to what extent it has been used to write a chapter or review. |

| 3.6. Obstacles related to the use of available evidence |
| --- |
| 3.6.2 . Inadequate synthesis of multiple bits of evidence.  Relevant evidence is available but consists of numerous bits of information that have not been randomized or interpreted. Evidence may be randomized but not systematically or rigorously. Conflicting evidence is presented without providing a definitive recommendation for the clinician who must make a decision. |
| 3.6.3. Difficulty applying results of randomized clinical trials to individual patients.  Clinical trials are often narrow in scope and may not apply to patients with comorbid conditions. |
| 4. Obstacles related to formulating the answer |
| 4.1. Failure to directly or completely answer the question.  Once the relevant information has been gathered, the searcher fails to directly or completely answer the doctor’s question (for example, owing to the inadequacy of available information or an inadequate synthesis of adequate information). |
| 4.2. Answer too long or too short.  The answer is too long to be helpful to a busy doctor or too short to completely address the information need. |
| 4.3. Answer directed at the wrong audience.  Answers for patients may not be helpful to doctors. |
| 4.4. Difficulty addressing unrecognised information needs apparent in the question.  It may not be clear how to address unrecognised information needs that are evident in the question. For example, the question might ask about the dose of a drug that is contraindicated (“What is the dose of tetracycline for acne in a pregnant woman?”). |
| 4.5. Discomfort of non-clinician searcher (for example, librarian) formulating an answer to be used in patient care.  Intermediary searchers who are not doctors (librarians, nurses) may be comfortable providing information on a given topic but not formulating an answer that would direct patient care. |
| 5. Obstacles related to using the answer to direct patient care |
| 5.1. Answer not trusted.  A seemingly adequate answer may not be used if the doctor does not trust the source. |
| 5.2. Answer moot or no longer needed.  The answer may be moot or irrelevant because it came too late or the patient improved or got worse before the answer could be applied. |
| 5.3. Answer inadequate.  If the answer is thought to be inadequate by the doctor, it may not be used to direct patient care. |
