# Supplementary material for: Development of two shortened systematic review formats for clinicians
Source: Implement Sci. 2013 Jun 14;8:68. doi: 10.1186/1748-5908-8-68 (PMC3691647; doi:10.1186/1748-5908-8-68)
Supplement: Additional file 2 — Assessment criteria for heuristic evaluation: modified for analysis of print materials. [file 1748-5908-8-68-S2.docx]

**Additional File 2. Assessment criteria for heuristic evaluation: modified for analysis of print materials**

| **Usability Principle** | **Explanation** |
| --- | --- |
| Match between system and the real world | The system should speak the users' language, with words, phrases and concepts familiar to the user, rather than system-oriented terms. Follow real-world conventions, making information appear in a natural and logical order. |
| Consistency and standards | Users should not have to wonder whether different words, situations, or actions mean the same thing. |
| Error prevention | A careful design prevents a problem from occurring in the first place. |
| Recognition rather than recall | Minimize the user's memory load by making objects, actions, and options visible/clear. The user should not have to remember information from one part of the document to another. Instructions for use should be visible or easily retrievable whenever appropriate. |
| Aesthetic and minimalist design | Documents should not contain information which is irrelevant or rarely needed. Every extra unit of information in a dialogue competes with the relevant units of information and diminishes their relative visibility. |
| Help and documentation | Even though it is better if the system can be used without documentation, it may be necessary to provide help and documentation. Any such information should be easy to search, focused on the user's task, list concrete steps to be carried out, and not be too large. |
