# Supplementary material for: Development of two shortened systematic review formats for clinicians
Source: Implement Sci. 2013 Jun 14;8:68. doi: 10.1186/1748-5908-8-68 (PMC3691647; doi:10.1186/1748-5908-8-68)
Supplement: Additional file 3 — Mapping exercise: Obstacles and descriptions of how they were addressed [53,54]. [file 1748-5908-8-68-S3.docx]

**Additional File 3.** **Mapping exercise: Obstacles and descriptions of how they were addressed**

| **Obstacles related to: Searching for relevant information** | | |
| --- | --- | --- |
| **Obstacle** | | **Assessment** |
| 1 | Topic or relevant aspect of topic not included in a resource that should logically include it | **Evidence-Expertise Format** |
|  |  | *Obstacle addressed by:*   - ‘Rosacea’ and ‘treatment’ are in the title to indicate clinical topic and orientation - Tables only present information on various treatments   **OUTSTANDING ISSUE**  The objective or a question could be added to help clarify focus of the document  **Action:** No action was taken as the other elements were felt to address the obstacle adequately |
|  |  | **Case–Based Format** |
|  |  | *Obstacle addressed by:*   - The words ‘rosacea’ and ‘treatment’ are in the title to indicate clinical topic and orientation - The objective clarifies that the focus of the document is treatment of rosacea - Table presents information related to treatments |
| 2 | Inadequacy of the resource's index | **Evidence-Expertise & Case-Based Formats** |
|  |  | Not applicable to these documents.  *Reason:* Each document is a maximum of 2 pages in length making an index inappropriate |
| 3 | Resource poorly organized | **Evidence-Expertise Format** |
|  |  | *Obstacle addressed by:*   - A table format is used: headings are used within the table to organize and highlight information   **Supportive Evidence**  The use of headings:   - aids search and recall [49] - has a greater effect if there is prior knowledge of the topic [50] |

| **Obstacles related to: Searching for relevant information** | | |
| --- | --- | --- |
| **Obstacle** | | **Assessment** |
| 3 | Resource poorly organized cont’d | **Case–Based Format** |
|  |  | *Obstacle addressed by:*   - Titles and headings are used to organize and highlight information - Titles specifically identify and make a distinction between the case study, synopsis, and table   **Supportive Evidence**  The use of titles:   - aids recall of what the text is about [51] - concrete titles improve recall, comprehension, and interest in comparison to abstract ones [52] |
| 4 | Resource not clinically oriented | **Evidence-Expertise & Case-Based Formats** |
|  |  | *Obstacle addressed by:*   - The words ‘rosacea’ and ‘treatment’ are in the title to indicate a clinical topic and orientation - The tables (for both formats) present information related to various treatments |
| 5 | Resource not authoritative or not trusted | **Evidence-Expertise Format** |
|  |  | *Obstacle addressed by:*   - Key issues of the quality of methodology are reported (e.g. quality assessment, limitations)     **OUTSTANDING ISSUE**  List full citation somewhere within the document in order to identify journal and authors, and for transparency  **Action:** Full citation was listed in the masthead |
|  |  | **Case-Based Format** |
|  |  | *Obstacle addressed by:*   - Full citation is listed at the top of the document in order to identify journal and authors, and for transparency - Key issues of the quality of methodology are reported (e.g. quality assessment, limitations) |

| **Obstacles related to: Searching for relevant information** | | |
| --- | --- | --- |
| **Obstacle** | | **Assessment** |
| 6 | Resource not current | **Evidence-Expertise & Case-Based Formats** |
|  |  | *Obstacle addressed by:*   - The review was published within the last 5 years *Due to the goal of brevity, the date of publication is reported (not the date the literature search was conducted).* |
| 7 | Inability to interact with a general resource as one could with a human resource | **Evidence-Expertise & Case-Based Formats** |
|  |  | Not applicable for these documents  *Reason:* Both formats are intended to be static documents |
| 8 | Incorrect information | **Evidence-Expertise & Case-Based Formats** |
|  |  | *Obstacle addressed by:*  The use of multiple measures safeguards against incorrect information,   - The rigorous methods of a systematic review provide a security against incorrect information [2] - The full-length review originated from a Cochrane review, then published in the Journal of American Academy of Dermatology thus it has gone through a peer-review process twice - Both shortened formats will go through a clinical content review |
| 9 | Information not current | **Evidence-Expertise Format** |
|  |  | **OUTSTANDING ISSUE**  The citation should be added so the date of the systematic review is available  **Action:** Full citation including the date was listed in the masthead |
|  |  | **Case-Based Format** |
|  |  | *Obstacle addressed by:*   - The date of the systematic review is listed within the citation *Due to the goal of brevity, the date of publication is reported (not the date the literature search was conducted).* |
| 10 | Failure to anticipate ancillary information needs | **Evidence-Expertise & Case-Based Formats** |
|  |  | Not applicable  *Reason:* A systematic review asks a specific question and answers it with the current available evidence thus by design does not include ancillary information [2] |
| **Obstacles related to: Searching for relevant information** | | |
| **Obstacle** | | **Assessment** |
| 11 | Failure to address common comorbid conditions | **Evidence-Expertise & Case-Based Formats** |
|  |  | Not applicable for these documents  *Reason:* It is not possible to address comorbid conditions unless the trials within the systematic review provided this information |
| 12 | Inadequate differential diagnosis | **Evidence-Expertise & Case-Based Formats** |
|  |  | Not applicable for these documents  *Reason:* The focus of the systematic review is therapy, not diagnosis |
| 13 | Failure to define important terms | **Evidence-Expertise & Case-Based Formats** |
|  |  | **OUTSTANDING ISSUE**  Consider defining some terms within the document  **ACTION:** Statistical definitions added to case-based format |
| 14 | Inadequate description of clinical procedures | **Evidence-Expertise & Case-Based Formats** |
|  |  | Not applicable for these documents  *Reason:* Therapy for this review does not require the performance of any clinical procedures on the part of the clinician |
| 15 | Vague or tangential information | **Evidence-Expertise & Case-Based Formats** |
|  |  | *Obstacle addressed by:*   - A systematic review asks a specific question and answers it with the current available evidence thus by design does not include tangential information [2] - The use of the two formats provide efforts to address vague information: the case-based format by contextualizing information, and the evidence-expertise format by integrating clinical expertise |

| **Obstacles related to: Searching for relevant information** | | |
| --- | --- | --- |
| **Obstacle** | | **Assessment** |
| 16 | Unnecessarily cautious writing style | **Evidence-Expertise Format** |
|  |  | *Obstacle addressed by:*   - Summary of Findings and tables use active voice with specific statements and concrete phrases - The prototype was scanned to ensure unnecessary hedge words (such as, can or may) were not used [53]   **Supportive Evidence**   - Text is easier to understand when an active voice is used rather than passive voice [49, 52, 54] - Concrete phrasing improves recall, comprehension, and interest [52] |
|  |  | **Case-Based Format** |
|  |  | Obstacle addressed by:   - Clinical Bottom Line and table uses active voice with specific statements and concrete phrases - The prototype was scanned to ensure unnecessary hedge words (such as, can or may) were not used [53]   **Supportive Evidence**   - Text is easier to understand when an active voice is used rather than passive voice [49, 52, 54] - Concrete phrasing improves recall, comprehension, and interest [52] |
| 17 | Tertiary care approach to primary care problem | **Evidence-Expertise & Case-Based Formats** |
|  |  | *Obstacle addressed by:*   - Therapies examined specifically relate to treatment of rosacea |
| 18 | Biased information due to conflicts of interest | **Evidence-Expertise & Case-Based Formats** |
|  |  | *Obstacle addressed by:*   - Authors declare no conflicts or competing interests |
| 19 | Failure to address the clinical question | **Evidence-Expertise & Case-Based Formats** |
|  |  | *Obstacle addressed by:*   - By design, a systematic review asks a specific question and answers it with the current available evidence [2] |

| **Obstacles related to: Searching for relevant information** | | |
| --- | --- | --- |
| **Obstacle** | | **Assessment** |
| 20 | Failure to study the comparison of interest | **Evidence-Expertise & Case-Based Formats** |
|  |  | *Obstacle addressed by:*   - The use of placebos is reported |
| 21 | Failure to study the outcome of interest | **Evidence-Expertise & Case-Based Formats** |
|  |  | *Obstacle addressed by:*   - By design, a systematic review asks a specific question and answers it with the current available evidence [2] |
| 22 | Failure to study the population of interest | **Evidence-Expertise & Case-Based Formats** |
|  |  | *Obstacle addressed by:*   - Methods and results section states the population as people with moderate to severe rosacea |
| 23 | Evidence based on flawed methods | **Evidence-Expertise & Case-Based Formats** |
|  |  | *Obstacle addressed by:*   - The full-length review originated from a Cochrane review with rigorous and thorough methods - Quality assessment was completed and reported on in the review |
| 24 | Failure to cite or include relevant evidence | **Evidence-Expertise & Case-Based Formats** |
|  |  | *Obstacle addressed by:*   - An exhaustive search is documented and executed in order to identify all relevant evidence as part of the methods for the review |
| 25 | Inadequate synthesis of multiple bits of evidence | **Evidence-Expertise & Case-Based Formats** |
|  |  | - *Focus groups (not described in this paper) will be used as the next step to identify if this has been done* |
| 26 | Difficulty applying results of randomized clinical trials to individual patients*.* | **Evidence-Expertise & Case-Based Formats** |
|  |  | Not applicable  *Reason:* A clinician would not be accessing these documents unless specifically interested in the question examined in the review |

| **Obstacles related to: Formulating an answer** | | | |
| --- | --- | --- | --- |
| **Obstacle** | | | **Assessment** |
| 27 | | Failure to directly or completely answer the question | **Evidence-Expertise & Case-Based Formats** |
|  |  |  | *Obstacle addressed by:*   - By design, a systematic review asks a specific question and answers it with the current available evidence [2] |
| 28 | | Answer too long or too short | **Evidence-Expertise & Case-Based Formats** |
|  |  |  | Not applicable  *Reason:* The focus of the study is to produce succinct shortened formats |
| 29 | | Answer directed at the wrong audience | **Evidence-Expertise & Case-Based Formats** |
|  |  |  | *Obstacle addressed by:*   - The review is intended for use by primary care physicians and chosen through a process used by the McMaster University Health Information Unit. These reviews were identified by primary care physicians as highly relevant [14] - The focus groups following this exercise will also serve to identify if the answer is not appropriately directed |
| 30 | | Difficulty addressing unrecognised information needs apparent in the question | **Evidence-Expertise & Case-Based Formats** |
|  |  |  | *Obstacle addressed by:*   - This refers to the question being posed by the clinician and there are few strategies guaranteed to identify unrecognized information needs - The approach used is to list out all adverse events |
| 31 | Answer not trusted | | **Evidence-Expertise Format** |
|  |  |  | **OUTSTANDING ISSUE**  List full citation somewhere within the document in order to identify journal and authors. This transparency allows the user to make a judgment about whether they feel the source is trustworthy  **Action:** Full citation was listed in the masthead |
|  |  |  | **Case-Based Format** |
|  |  |  | *Obstacle addressed by:*   - Full citation is listed at the top of the document in order to identify journal and authors, and for transparency |

| **Obstacles related to: Using the answer to direct patient care** | | |
| --- | --- | --- |
| **Obstacle** | | **Assessment** |
| 32 | Answer inadequate | **Evidence-Expertise & Case-Based Formats** |
|  |  | *Obstacle addressed by:*   - An exhaustive search is documented and executed in order to identify all relevant evidence as part of the methods for the review - The use of the two formats provide efforts to completely answer the question: the case-based format by contextualizing information, and the evidence-expertise format by integrating clinical expertise |

Obstacle column derived from: Ely JW, Osheroff JA, Ebell MH, Chambliss ML, Vinson DC, Stevermer JJ, Pifer EA. BMJ. 2002;324(7339):710.
