# Supplementary material for: Development of two shortened systematic review formats for clinicians
Source: Implement Sci. 2013 Jun 14;8:68. doi: 10.1186/1748-5908-8-68 (PMC3691647; doi:10.1186/1748-5908-8-68)
Supplement: Additional file 4 — Shortened format: Initial versions of two prototypes. [file 1748-5908-8-68-S4.pdf]

Additional File 4. Shortened format: Initial versions of two prototypes

[Case Based]

Systematic review of rosacea treatments

---

van Zuuren EJ, Gupta AK, Gover MD, Graber M, Hollis S. Systematic review of rosacea treatments. J Am Acad Dermatol. 2007 Jan;56(1):107-15.

CASE STUDY

Background Rosacea is a common chronic skin and ocular condition.

Case Presentation A 35-year-old woman had developed moderate swelling, erythema and papules of the central part of her face for 8 weeks. She had been applying various topical cosmetic products sold for acne with no change in her condition. One of her hobbies is hiking and she noticed sun exposure aggravated her skin condition, also resulting in burning and stinging sensations.

Treatment The patient consulted her general practitioner who prescribed prednicarbat cream for topical application on the affected regions. She observed a slight improvement of the skin condition during the first week, but suddenly developed a severe worsening with erythema, papules and many pustules later on.

She presented to a dermatologist and was diagnosed with "steroid rosacea". She went off the steroid, started topical treatment with metronidazole 1% and oral treatment with metronidazole 500 mg twice daily for 2 weeks.

Outcome & Follow-up After an initial worsening during the first 3 days the skin condition improved. The topical treatment was continued twice daily for 4 weeks and then reduced to once daily for an additional 4 weeks. As well, sun screen was applied whenever outdoors. She continued intermittent topical use of metronidazole 1% and remained free of symptoms except for sporadic slight centrofacial erythema.

Clinical Bottom Line

- There is evidence that topical metronidazole and azelaic acid are effective ❶❷❸❹○
- There is some evidence that oral metronidazole and tetracycline are effective ❶❷❸○○

Strength of Evidence Rating

The strength of evidence ratings are based on the overall quantity and quality of clinical evidence.

STRONG RESEARCH SUPPORT	❶❷❸❹❺
MODERATE RESEARCH SUPPORT	❶❷❸❹○
WEAK RESEARCH SUPPORT	❶❷❸○○
STRONG EXPERT OPINION	❶❷○○○
WEAK EXPERT OPINION	❶○○○○

SYSTEMATIC REVIEW SYNOPSIS

This systematic review sought to assess the evidence for the efficacy and safety of rosacea therapies.

Methods Multiple databases were systematically searched. Randomized controlled trials in people with moderate to severe rosacea were included. Study selection, assessment of methodologic quality, data extraction, and analysis were carried out by two independent researchers.

Results In all, 29 studies met inclusion criteria. Topical metronidazole is more effective than placebo (odds ratio 5.96, 95% confidence interval 2.95 - 12.06). Azelaic acid is more effective than placebo (odds ratio 2.45, 95% confidence interval 1.82 - 3.28). Firm conclusions could not be drawn about other therapies. See **Table 1**.

Limitations The quality of the studies was generally poor.

Table 1. Rosacea Therapies

Signs / Symptoms	Treatment	Issues (if any)	Meta-analysis
Limited number of papules / pustules	Topical therapies<br>▪ Metronidazole (0.75%, 1%)<br>▪ Clindamycin lotion<br>▪ Permethrin 5% cream<br>▪ Tretinoin cream<br>▪ Sulfacetamide 10%/sulfur 5%<br>▪ Azelaic acid (15% gel, 20% cream)		Topical metronidazole and placebo<br>OR: 5.96, 95% CI: 2.96, 12.06<br>**Level of Evidence**<br>❶❷❸❹○<br>Topical azelaic acid and placebo<br>OR: 2.45, 95% CI: 1.82, 3.28<br>**Level of Evidence**<br>❶❷❸❹○
	Proposed therapies<br>▪ Tacrolimus<br>▪ Topical NADH	NADH, reduced form of ß-nicotinamide adenine inucleotide	
More extensive skin lesions	Oral antibiotics<br>▪ Tetracycline<br>▪ Ampicillin<br>▪ Metronidazole<br>▪ Erythromycin	Possible side effects include:<br>▪ gastrointestinal symptoms<br>▪ photosensitivity<br>▪ candidal vaginitis<br>▪ reduction in oral contraceptive<br>▪ efficacy	Oral tetracycline and placebo<br>OR: 2.59, 95% CI: 0.70, 9.64<br>**Level of Evidence**<br>❶❷❸○○
	Oral / topical therapy combination	▪ Discontinue oral treatment once sufficient efficacy noted<br>▪ Maintenance therapy with topical medications	
Vascular symptoms	Pulse dye laser, intense pulsed light<br>**Level of Evidence**<br>❶❷❸○○		
Severe or persistent rosacea	Oral isotretinoin<br>▪ 13-cis-retinoic acid<br>**Level of Evidence**<br>❶❷❸○○	Possible side effects include:<br>▪ dry sensitive skin<br>▪ dry mucosae<br>▪ dry eyes<br>▪ pruritis<br>▪ dermatitis<br>▪ myalgia<br>▪ elevated liver enzymes<br>▪ cholesterol and triglyceride elevation<br>Routine monitoring of liver functions, cholesterol, triglycerides required<br>Possible fetal abnormalities for women who become pregnant	
Control of flushing	Oral hypotensives<br>▪ Clonidine Rilmenidine<br>**Level of Evidence**<br>❶❷❸○○		
Rhinophyma	Oral Low-dose isotretinoin<br>Laser therapy<br>Surgical intervention<br>**Level of Evidence**<br>❶❷❸○○		
Ocular rosacea	Oral antibiotics: Tetracycline<br>Topicals Metronidazole<br>Fusidic acid gel<br>**Level of Evidence**<br>❶❷❸○○		

Learning Points & Take Home Messages

- There is evidence that topical metronidazole and azelaic acid are effective.
- There is some evidence that oral metronidazole and tetracycline are effective.
- More well-designed, randomized controlled trials are required to provide better evidence of the efficacy and safety of other rosacea therapies.

Case study adapted from Case report no. 1: http://rosacea.dermis.net/content/e05quizcase/index_eng.html

[Evidence-Expertise]

Systematic review of rosacea treatments

Van Zuuren EJ, Gupta AK, Gover MD, Graber M, Hollis S.

Summary of Findings

In all, 29 studies met inclusion criteria. Topical metronidazole is more effective than placebo (odds ratio 5.96, 95% confidence interval 2.95-12.06). Azelaic acid is more effective than placebo (odds ratio 2.45, 95% confidence interval 1.82-3.28). Firm conclusions could not be drawn about other therapies.

There is evidence that topical metronidazole and azelaic acid are effective. There is some evidence that oral metronidazole and tetracycline are effective. More well-designed, randomized controlled trials are required to provide better evidence of the efficacy and safety of other rosacea therapies. (J Am Acad Dermatol 2007;56:107-15.)

Limitations of Study

The quality of the studies was generally poor.

Expert Clinician Interpretation:

Expert Interpretation:
- Topical metronidazole cream (0.75% and 1%) and azelaic acid cream (15 and 20%) are effective and safe for short term use (8 to 12 wks)
- Oral oxytetracycline (dose = (need from original study)) may be effective for ocular rosacea. Oral tetracycline is more effective than placebo for physician assessment but not for patient outcomes.
- There is no evidence for use of other agents including dapsone, erythromycin, topical tretinoin, benzoyl peroxide, diet or sun protection. The quality of reported studies is poor with lack of blinding, allocation concealment, and intention to treat analysis.

Treatment	Period	Self Assessed Improvement of Severity	Physician's evaluation	Adverse Events
**Symptom**: Limited number of papules/pustules				
Topical metronidazole ❶❷❸❹○	8-9 weeks	Better than placebo: OR 7.0; 95% CI 2.5 - 20.0	Better than placebo: OR 7.01; 95% CI 3.56 - 13.81	Mild: pruritus, skin irritation, dry skin
Azelaic acid ❶❷❸❹○	9 - 12 weeks	Improvement in azelaic acid group. Split-face, within-patient study confirmed results (marginal OR 30.1; P<.0003)		Side effects were considered mild and transient with burning, stinging, and irritation being reported most frequently. More side effects in azelaic group(11.5%) versus placebo group (5.7%) (OR 1.61; 95% CI 0.89 - 2.92)
Metronidazole plus sunscreen SPF 15 ❶❷❸○○		Poorly designed study favoured metronidazole plus sunscreen over placebo		
Topical azelaic acid ❶❷❸○○	15 weeks	No significant difference with topical metronidazole	Rated azelaic acid more improved (OR 1.84; 95% CI 1.10 - 3.09)	Mild to moderate, mostly transient: burning, stinging, irritation
Topical Permethrin Level of evidence: ❶❷❸○○		Inferior to topical metronidazole		
Sodium sulfacetamide 10%/sulfur 5% ❶❷❸○○		90% of patients stated improvement from sodium sulfacetamide versus 58% in placebo group (P <.001).	Physicians found improvement from sodium sulfacetamide 10%/sulfur 5%	Dryness, erthema, pruritus
Clarithromycin and omeprazole ❶❷❸○○		Impossible to draw conclusions from data		

Treatment	Period	Self Assessed Improvement of Severity	Physician’ s evaluation	Adverse Events
**Symptom**: More extensive skin lesions				
Oral Tetracycline<br>❶❷❸○○	8 week	No significant difference with topical metronidazole		
	6 weeks	14/20 treated with tetracycline considered improved versus 14/17 treated with ampicillin (OR 0.50; 95% CI 0.10 - 2.41)	17/20 treated with tetracycline considered improved versus 9/17 treated with ampicillin (OR 5.04; 95% CI 1.07 - 23.82)	Mild and transient: 3/17 in ampicillin group reported adverse effects vs 1/20 in tetracycline group
Tetracycline<br>❶❷❸○○	4-6 weeks	Insufficient evidence according to patient assessment.	Physican assessment found tetracyclines more effective than placebo (OR 6.06; 95% CI 2.96 - 12.42)	
Oral metronidazole and topical hydrocortisone 1% cream<br>❶❷❸○○			Considered 10 of 14 participants improved versus 2 of 13 on placebo (OR 13.75; 95% CI 2.05 - 92.04)	
Ampicillin<br>❶❷❸○○	6 weeks	Favoured ampicillin over placebo (OR 5.19; 95% CI 1.11 - 24.14)	Favoured ampicillin over placebo, but not statistically significant. (OR 4.22; 95% CI 0.98 - 18.12)	Mild and transient
Oral oxytetracycline	12 weeks	One study found no statistical difference with oral metronidazole		None reported
Benzoyl peroxide<br>❶❷❸○○	4 weeks		Improvement on physician’ s global evaluation compared with placebo (OR 3.17; 95% CI 1.08 - 9.31)	Adverse effects include irritation and burning.
Benzoyl peroxide 5%/clindamycin 1% gel<br>❶❷❸○○	12 weeks	Patient’ s global assessment better in benzoyl peroxide and clindamycin group (1.54 - much to slightly better) versus placebo group (2.5 - slightly better to same)(P = .0002)	Physician’ s global assessment better in benzoyl peroxide and clindamycin group (1.85 - marked to definite improvement) versus placebo group (2.96 - minimal improvement) (P=.0026)	Adverse effects included site burning and itching.
Rilmenidine<br>❶❷❸○○		No significant difference		
Oral metronidazole<br>❶❷❸○○	12 weeks			None reported
**Symptom**: Flushing				
Rilmenidine<br>❶❷❸○○		No significant difference		
**Symptom**: Ocular Rosacea				
Tetracycline<br>❶❷❸○○	4-6 weeks	Insufficient evidence according to patient assessment.	Physican assessment found tetracyclines more effective than placebo (OR 6.06; 95% CI 2.96 - 12.42)	
**Other**				
Benzoyl peroxide 5%/erthromycin 3% gel<br>❶❷❸○○	4 weeks	no significant difference with metronidazole gel (OR 0.92; 95% CI 0.21 - 4.11)		
