# Supplementary material for: Development of two shortened systematic review formats for clinicians
Source: Implement Sci. 2013 Jun 14;8:68. doi: 10.1186/1748-5908-8-68 (PMC3691647; doi:10.1186/1748-5908-8-68)
Supplement: Additional file 5 — Shortened format: Revised versions of two prototypes. [file 1748-5908-8-68-S5.pdf]

Figure 3. Shortened format: Revised versions of two prototypes

[Case Based]

Systematic review of rosacea treatments

van Zuuren EJ, Gupta AK, Gover MD, Graber M, Hollis S. Systematic review of rosacea treatments. J Am Acad Dermatol. 2007 Jan;56(1):107-15.

CASE STUDY

Case Presentation

- A 35-year-old woman had developed moderate swelling, erythema and papules of the central part of her face for 8 weeks.
- She had been applying various topical cosmetic products sold for acne with no change in her condition.
- One of her hobbies is hiking and she noticed sun exposure aggravated her skin condition, also resulting in burning and stinging sensations.

Clinical Bottom Line

- Topical metronidazole and azelaic acid are effective ❶❷❸❹○
- There is some evidence that oral metronidazole and tetracycline are effective ❶❷❸○○

Treatment

- The patient consulted her family physician who prescribed topical treatment with metronidazole 1% and oral treatment with metronidazole 500 mg twice daily for 2 weeks.

Outcome & Follow-up

- After an initial worsening during the first 3 days the skin condition improved.
- The topical treatment was continued twice daily for 4 weeks and then reduced to once daily for an additional 4 weeks. As well, sun screen was applied whenever outdoors.
- She continued intermittent topical use of metronidazole 1% and remained free of symptoms except for sporadic slight centrofacial erythema.

SYSTEMATIC REVIEW SYNOPSIS

Purpose To assess the evidence for the efficacy and safety of rosacea therapies.

Methods

Multiple databases were systematically searched. Randomized controlled trials in people with moderate to severe rosacea were included. Study selection, assessment of methodologic quality, data extraction, and analysis were carried out by two independent researchers. The outcome measure was participant-assessed changes in rosacea severity

Results

- 29 studies met the inclusion criteria.
- Topical metronidazole is more effective than placebo (odds ratio 5.96, 95% confidence interval 2.95 - 12.06).
- Azelaic acid is more effective than placebo (odds ratio 2.45, 95% confidence interval 1.82 - 3.28).
- Firm conclusions could not be drawn about other therapies.

Stats Tip Odds Ratio: A way of comparing whether the probability of an event is the same for 2 groups.
OR = 1: Event is equally likely in both groups
OR > 1: Event is more likely in the 1st group

See **Table 1** (next page) for more information on each therapy.

Limitations Quality of the studies was generally poor.

Strength of Evidence Rating Scale

The strength of evidence ratings for this guide are based on the overall quantity and quality of clinical evidence.

STRONG RESEARCH SUPPORT ❶❷❸❹❺
The results are consistent from high quality studies. The conclusions are unlikely to change with further research.

MODERATE RESEARCH SUPPORT ❶❷❸❹○
Current research supports the findings. However, it is possible that conclusions could change with further research.

WEAK RESEARCH SUPPORT ❶❷❸○○
There are limited studies available for drawing conclusions, or the existing studies have significant limitations.

STRONG EXPERT OPINION ❶❷○○○
Consensus based on a panel of experts in the topic field.

WEAK EXPERT OPINION ❶○○○○
No research-based evidence.

Table 1. Rosacea Therapies

Symptom	Treatments	Adverse Events	Meta-analysis
Limited number of papules / pustules	Topical therapies<br>▪ Metronidazole (0.75%, 1%)<br>▪ Clindamycin lotion<br>▪ Permethrin 5% cream<br>▪ Tretinoin cream<br>▪ Sulfacetamide 10%/sulfur 5%<br>▪ Azelaic acid (15% gel, 20% cream)		Topical metronidazole vs placebo<br>OR: 5.96, 95% CI: 2.96, 12.06<br>**Strength of Evidence**<br>❶❷❸❹○ (2 trials)<br>Topical azelaic acid vs placebo<br>OR: 2.45, 95% CI: 1.82, 3.28<br>**Strength of Evidence**<br>❶❷❸❹○ (3 trials)
	Proposed therapies<br>▪ Tacrolimus<br>▪ Topical NADH	NADH, reduced form of ß-nicotinamide adenine inucleotide	
More extensive skin lesions	Oral antibiotics<br>▪ Tetracycline<br>▪ Ampicillin<br>▪ Metronidazole<br>▪ Erythromycin	Possible side effects include:<br>▪ gastrointestinal symptoms<br>▪ photosensitivity<br>▪ candidal vaginitis<br>▪ reduction in oral contraceptive<br>▪ efficacy	Oral tetracycline vs placebo<br>OR: 2.59, 95% CI: 0.70, 9.64<br>**Strength of Evidence**<br>❶❷❸○○ (2 trials)
	Oral / topical therapy combination	▪ Discontinue oral treatment once sufficient efficacy noted<br>▪ Maintenance therapy with topical medications	*No randomized controlled trials available for evaluation.*
Vascular symptoms	Pulse dye laser, intense pulsed light		*No randomized controlled trials available for evaluation.*
Severe or persistent rosacea	Oral isotretinoin<br>▪ 13-cis-retinoic acid	Possible side effects include:<br>▪ dry sensitive skin<br>▪ dry mucosae<br>▪ dry eyes<br>▪ pruritis<br>▪ dermatitis<br>▪ myalgia<br>▪ elevated liver enzymes<br>▪ cholesterol and triglyceride elevation<br>Routine monitoring of liver functions, cholesterol, triglycerides required<br>Possible fetal abnormalities for women who become pregnant	*No randomized controlled trials available for evaluation.*
Control of flushing	Oral hypotensives<br>▪ Clonidine<br>▪ Rilmenidine<br>**Strength of Evidence**<br>❶❷❸○○<br>(1 trial: Rilmenidine)		
Rhinophyma	Oral: Low-dose isotretinoin<br>Laser therapy<br>Surgical intervention		*No randomized controlled trials available for evaluation.*
Ocular rosacea	Oral antibiotics: Tetracycline<br>Topicals Metronidazole<br>Fusidic acid gel<br>**Strength of Evidence**<br>❶❷❸○○<br>(2 trials: 1. Metronidazole ; 2. Oxytetracycline)		

Systematic review of rosacea treatments

van Zuuren EJ, Gupta AK, Gover MD, Graber M, Hollis S. Systematic review of rosacea treatments. J Am Acad Dermatol. 2007 Jan;56(1):107-15.

Expert Interpretation:
- Topical metronidazole cream (0.75% and 1%) and azelaic acid cream (15 and 20%) are effective and safe for short term use (8 to 12 wks)
- Oral oxytetracycline (dose = (need from original study)) may be effective for ocular rosacea. Oral tetracycline is more effective than placebo for physician assessment but not for patient outcomes.
- There is no evidence for use of other agents including dapsone, erythromycin, topical tretinoin, benzoyl peroxide, diet or sun protection. The quality of reported studies is poor with lack of blinding, allocation concealment, and intention to treat analysis.

In all, 29 studies met inclusion criteria. Topical metronidazole is more effective than placebo (odds ratio 5.96, 95% confidence interval 2.95-12.06). Azelaic acid is more effective than placebo (odds ratio 2.45, 95% confidence interval 1.82-3.28). Firm conclusions could not be drawn about other therapies.
There is evidence that topical metronidazole and azelaic acid are effective. There is some evidence that oral metronidazole and tetracycline are effective. More well-designed, randomized controlled trials are required to provide better evidence of the efficacy and safety of other rosacea therapies. (J Am Acad Dermatol 2007;56:107-15.)

Methodology: Reviewed all RCTs evaluating any type of intervention used to treat rosacea. Study participants had to be older than 19 years with moderate to severe rosacea as assessed by a physician. Two reviewers determined study quality based on a set of quality assessment criteria. 29 RCTS were included, 8 were classified as high quality and 21 as intermediate.

Limitations: The quality of the studies was generally poor.

Strength of Evidence Rating Scale
The strength of evidence ratings for this guide are based on the overall quantity and quality of clinical evidence.

Strong Research Support
❶❷❸❹❺
The results are consistent with high quality studies. The conclusions are unlikely to change with further research.

Moderate Research Support
❶❷❸❹●
Current research supports the findings. However, it is possible that conclusions could change with further research.

Weak Research Support ❶❷❸●●
There are limited studies available for drawing conclusions, or the existing studies have significant limitations.

Strong Expert Opinion ❶❷●●●
Consensus based on a panel of experts in the topic field.

Weak Expert Opinion ❶●●●●
No research-based evidence.

Treatment	Follow Up	Outcomes		
		Self Assessed Improvement	Physician's Evaluation	Adverse Events
**Symptom: Limited number of papules/pustules**				
Topical metronidazole (9 trials) ❶❷❸❹●	8-9 weeks	Better than placebo: OR 7.0; 95% CI 2.5 - 20.0	Better than placebo: OR 7.01; 95% CI 3.56 - 13.81	Mild: pruritus, skin irritation, dry skin
Azelaic Acid (4 trials) ❶❷❸❹●	9-12 weeks	Improvement in azelaic acid group. Split-face, within-patient study confirmed results (marginal OR 30.1; P<.0003)		Side effects were considered mild and transient with burning, stinging, and irritation being reported most frequently. More side effects in azelaic group (11.5%) versus placebo group (5.7%) (OR 1.61; 95% CI 0.89 - 2.92)
Metronidazole plus sunscreen SPF 15 (1 trial) ❶❷❸●●	n/a	Poorly designed study favoured metronidazole plus sunscreen over placebo		n/a
Topical azelaic acid (2 trials) ❶❷❸●●	15 weeks	No significant difference with topical metronidazole	Rated azelaic acid more improved (OR 1.84; 95% CI 1.10 - 3.09)	Mild to moderate, mostly transient: burning, stinging, irritation
Topical Permethrin (1 trial) ❶❷❸●●	n/a	Inferior to topical metronidazole	n/a	n/a
Sodium sulfacetamide 10%/ sulfur 5% (1 trial) ❶❷❸●●	n/a	90% of patients stated improvement from sodium sulfacetamide versus 58% in placebo group (P <.001).	Physicians found improvement from sodium sulfacetamide 10%/sulfur 5%	Dryness, erthema, pruritus
Clarithromycin and omeprazole (1 trial) ❶❷❸●●	n/a	Impossible to draw conclusions from data		n/a

van Zuuren EJ, Gupta AK, Gover MD, Graber M, Hollis S. Systematic review of rosacea treatments. J Am Acad Dermatol. 2007 Jan;56(1):107-15.

Treatment	Follow Up	Outcomes		
		Self Assessed Improvement	Physician's Evaluation	Adverse Events
**Symptom: More extensive skin lesions**				
Oral Tetracycline (2 trials) ❶❷❸●●	8 weeks	No significant different with topical metronidazole		n/a
	8 weeks	14/20 treated with tetracycline considered improved versus 14/17 treated with ampicillin (OR 0.50; 95% CI 0.10 - 2.41)	17/20 treated with tetracycline considered improved versus 9/17 treated with ampicillin (OR 5.04; 95% CI 1.07 - 23.82)	Mild and transient: 3/17 in ampicillin group reported adverse effects vs 1/20 in tetracycline group
Tetracycline (3 trials) ❶❷❸●●	4-6 weeks	Insufficient evidence according to patient assessment.	Physican assessment found tetracyclines more effective than placebo (OR 6.06; 95% CI 2.96 - 12.42)	n/a
Oral metronidazole and topical hydrocortisone 1% cream (1 trial) ❶❷❸●●	n/a	n/a	Considered 10 of 14 participants improved versus 2 of 13 on placebo (OR 13.75; 95% CI 2.05 - 92.04)	n/a
Ampicillin (1 trial) ❶❷❸●●	6 weeks	Favoured ampicillin over placebo (OR 5.19; 95% CI 1.11 - 24.14)	Favoured ampicillin over placebo, but not statistically significant. (OR 4.22; 95% CI 0.98 - 18.12)	Mild and transient
Benzoyl peroxide (1 trial) ❶❷❸●●	4 weeks	n/a	Improvement on physician's global evaluation compared with placebo (OR 3.17; 95% CI 1.08 - 9.31)	Adverse effects include irritation and burning.
Oral oxytetracycline (1 trial)	12 weeks	One study found no statistical difference with oral metronidazole		None reported
Benzoyl peroxide 5%/clindamycin 1% gel (1 trial) ❶❷❸●●	12 weeks	Patient's global assessment better in benzoyl peroxide and clindamycin group (1.54 - much to slightly better) versus placebo group (2.5 - slightly better to same)(P = .0002)	Physician's global assessment better in benzoyl peroxide and clindamycin group (1.85 - marked to definite improvement) versus placebo group (2.96 - minimal improvement) (P=.0026)	Adverse effects included site burning and itching.
Rilmenidine (1 trial) ❶❷❸●●	n/a	No significant difference		n/a
Oral metronidazole (1 trial) ❶❷❸●●	12 weeks			None reported
**Symptom: Flushing**				
Rilmenidine (1 trial) ❶❷❸●●	n/a	No significant difference		n/a
**Symptom: Ocular Rosacea**				
Tetracycline (3 trials) ❶❷❸●●	4-6 weeks	Insufficient evidence according to patient assessment.	Physican assessment found tetracyclines more effective than placebo (OR 6.06; 95% CI 2.96 - 12.42)	n/a
**Symptom: Other**				
Benzoyl peroxide 5%/erthromycin 3% gel (1 trial) ❶❷❸●●	4 weeks	no significant difference with metronidazole gel (OR 0.92; 95% CI 0.21 - 4.11)	n/a	n/a
